# Quinpirole-Mediated Regulation of Dopamine D2 Receptors Inhibits Glial Cell-Induced Neuroinflammation in Cortex and Striatum after Brain Injury

**DOI:** 10.3390/biomedicines9010047

**Published:** 2021-01-07

**Authors:** Sayed Ibrar Alam, Min Gi Jo, Tae Ju Park, Rahat Ullah, Sareer Ahmad, Shafiq Ur Rehman, Myeong Ok Kim

**Affiliations:** 1 Division of Life Sciences and Applied Life Science (BK21 FOUR), College of Natural Science, Gyeongsang National University, Jinju 52828, Korea; ibrar@gnu.ac.kr (S.I.A.); mingi.cho@gnu.ac.kr (M.G.J.); Rahatullah1414@gnu.ac.kr (R.U.); sareer_50@gnu.ac.kr (S.A.); shafiq.qau.edu@gmail.com (S.U.R.); 2 Paul O’Gorman Leukaemia Research Centre, Institute of Cancer Sciences, MVLS, University of Glasgow, Glasgow G12 8QQ, UK; 2358860p@student.gla.ac.uk

**Keywords:** brain injury, quinpirole, dopamine D2 receptors, glial cell, neuroinflammation, neurodegeneration

## Abstract

Brain injury is a significant risk factor for chronic gliosis and neurodegenerative diseases. Currently, no treatment is available for neuroinflammation caused by the action of glial cells following brain injury. In this study, we investigated the quinpirole-mediated activation of dopamine D2 receptors (D2R) in a mouse model of traumatic brain injury (TBI). We also investigated the neuroprotective effects of quinpirole (a D2R agonist) against glial cell-induced neuroinflammation secondary to TBI in adult mice. After the brain injury, we injected quinpirole into the TBI mice at a dose of 1 mg/kg daily intraperitoneally for 7 days. Our results showed suppression of D2R expression and deregulation of downstream signaling molecules in ipsilateral cortex and striatum after TBI on day 7. Quinpirole administration regulated D2R expression and significantly reduced glial cell-induced neuroinflammation via the D2R/Akt/glycogen synthase kinase 3 beta (GSK3-β) signaling pathway after TBI. Quinpirole treatment concomitantly attenuated increase in glial cells, neuronal apoptosis, synaptic dysfunction, and regulated proteins associated with the blood–brain barrier, together with the recovery of lesion volume in the TBI mouse model. Additionally, our in vitro results confirmed that quinpirole reversed the microglial condition media complex-mediated deleterious effects and regulated D2R levels in HT22 cells. This study showed that quinpirole administration after TBI reduced secondary brain injury-induced glial cell activation and neuroinflammation via regulation of the D2R/Akt/GSK3-β signaling pathways. Our study suggests that quinpirole may be a safe therapeutic agent against TBI-induced neurodegeneration.

## 1. Introduction

Traumatic brain injury (TBI) is a global risk factor and the leading cause of neurological disability. Recent studies have reported that TBI is associated with several neurodegenerative diseases, such as Alzheimer’s and Parkinson’s disease [1,2,3]. TBI leads to a primary injury, which is followed by a secondary brain injury. Primary brain injury refers to the direct mechanical force applied at the time of the initial impact on the brain. Secondary brain injury occurs as a consequence of the initial traumatic events. It refers to the involvement of the brain vasculature as well as the blood–brain barrier (BBB) disruption, which results in significant complications in the brain [4]. Neuroinflammation is the principal hallmark of brain injury, followed by astrocyte and microglia activation and release of pro-inflammatory cytokines and chemokines, which impair the endogenous self-repair ability of the brain and eventually cause neuronal apoptosis and neurodegenerative conditions [5,6]. Several studies have proved that brain injuries precipitate an inflammatory response with activation of neuroinflammatory mediators [7]. Therefore, it is important to develop neuroprotective and neurorestorative agents to treat TBI. Notably, this subject offers much scope for extensive research to treat the TBI-induced neuroinflammatory response and inflammatory cytokine release. Restoration of BBB integrity and treatment of neuroinflammation is the key therapeutic goals in patients with brain injury-induced pathological events.

Dopamine (DA) is a major neurotransmitter that controls abnormal neuronal excitotoxicity and regulates the function of the dopaminergic system in the brain [6,8,9]. Dopamine D2 receptors (D2R) belong to the class of G protein-coupled receptors that are activated by DA and participate in essential functions, including innate immunity and neuroinflammatory responses [10,11]. However, previous studies have reported a significantly increased inflammatory response in D2R-knockout (D2R−/−) mice [12]. D2R is expressed in several regions of the brain, including the cerebral cortex, hippocampus, and striatum [13]. A previous study has shown that DA receptors are expressed on glial and immune cells [14]. Several studies have reported that DA plays a vital role in humans and animals and that cortical dopaminergic dysfunction is associated with attention deficit hyperactivity disorder [15,16]. A recent study has reported that cortical D2R is involved in psychotic and mood disorders and regulates neuronal circuits [17]. Deregulation of the DA system could be a significant contributor to behavioral and cognitive deficits that are observed after TBI. A growing body of evidence suggests that D2R agonists protect against neuroinflammation and immune reactions, perhaps by inhibiting cytokine release [18,19]. An earlier study reported that quinpirole-activated D2R positively affects neuronal activity in the cingulate cortex and striatum [20]. However, limited studies have reported the role of D2R activation in the inhibition of glial cell-induced neuroinflammatory responses following brain injury.

Akt, a serine-threonine kinase, is known to play an essential role in the cell death/survival pathway. Akt phosphorylates and inhibits several substrates, including glycogen synthase kinase 3 beta (GSK3-β) [21]. A previous study investigated the regulation of the Akt pathway by stimulation of DA receptors and reported possible regulation of the Akt/GSK3-β pathway via regulation of D2R [22].

In this study, we investigated the possible regulation of D2R in the cortical region of the brain, which is the primary target of brain injury, and also explored the striatal region in a TBI mouse model. Furthermore, we investigated the therapeutic potential of post-TBI administration of quinpirole hydrochloride [19,23]. We observed that quinpirole administration at a dose of 1 mg/kg could potentially protect against brain injury-induced gliosis, neuroinflammation, neurodegeneration, lesion volume, synaptic dysfunction and and regulated proteins associated with the BBB via stimulation of D2R, particularly in the ipsilateral cortex of TBI mice. We could also confirm microglial involvement in D2R deregulation and that quinpirole at a dose of 20 µM is sufficient to stimulate D2R and regulate Akt levels in neuronal cell lines. This study highlights that quinpirole administration ipsilateral side of TBI mouse brain stimulated D2R and lead to the recovery of brain function via regulation of the Akt/GSK3-β signaling pathway and inhibition of a glial cell-induced neuroinflammatory response.

## 2. Materials and Methods

### 2.1. Animals

Male wild-type C57BL/6N mice, 7 weeks of age with 25–30 g weight, were obtained from Samtako Bio Korea. The animals were acclimatized in the animal care center at Gyeongsang National University, South Korea. The animal were maintained in the control environment with 12/12 h light/dark cycle at 23 °C, and 60 ± 10% humidity with free access to food and water. The mice were randomly divided into following different groups; control, TBI, and TBI + quinpirole after a week of acclimatization. The animals were handled carefully according to the guidelines of the Institutional Animal Care and Use committee (IACUC) (5 March 2019. Approval ID: 125), Division of Life Science and Applied Life Science, Gyeongsang National University, Republic of South Korea.

### 2.2. Quinpirole Treatment for Mice

The treated animals were divided into the following groups:

Saline treated control group, Stab Wound Cortical Injury, Stab Wound Cortical Injury + quinpirole.

Quinpirole was dissolved in distilled water and administered daily intraperitoneally (i.p) at a dose of 1 mg/kg body weight for 7 days. For western blot (*n* = 5) and for confocal experiments (*n* = 6) mice per group were used. The chemical quinpirole was purchased from Tocris-Cookson (Bristol, UK).

### 2.3. Stab Wound Cortical Injury

The stab wound cortical brain injury mouse model was established as previously described with modification [24]. Briefly, the mice were anesthetized with Rompun (0.05 mL/100 g body weight) and Zoletil (0.1 mL/100 g body weight). The mice were placed on stereotaxic apparatus and the skull was exposed the by making a mid-longitudinal incision. The dental drill was used to make a circular craniotomy 4 mm in diameter (2 mm lateral to the midline and 1 mm posterior to the bregma) in the skull. For stab wound injury, a sharp edge scalpel blade was inserted (3 mm; right hemisphere) in the mouse brain and kept for 1 min in the brain and then removed slowly. The bone wax was applied to cover the rupture skull followed by stitching with a silk suture to close the wound area. Next, the animals were placed carefully by providing continuous heating with a heating lamp until fully recovered from anesthesia and proceeded for further experiments.

### 2.4. Protein Extraction

After the completion of the mice treatment, all the animals were first anesthetized and then sacrificed carefully. After the surgery brain were immediately collected and froze on dry ice. The ipsilateral cortex of TBI brain tissue was homogenized using PRO-PREP protein extract solution (iNtRON Biotechnology, Burlington, NJ, USA). to extract protein from tissues followed by centrifugation and stored at −80 °C. The samples were centrifuged at speed of 13,000× *g* rpm at 4 °C for 25 min. The supernatants were collected and stored at −80 °C for immunoblotting.

### 2.5. Western Blot Analysis

The western blot analysis was assessed as previously described with minor changes [25,26,27]. In brief, an equal volume of 20–30 μg of proteins (extracted from the ipsilateral cortex) was mixed with 2× Sample Buffer (Invitrogen). To separate the proteins, an equal volume of the proteins were run on 10% of SDS polyacrylamide gel electrophoresis and transferred to the PVDF membrane followed by blocking in 5% skim milk. The membranes were slightly washed to clear the skim milk. The primary antibody was incubated overnight at 4 °C 1:1000, anti-(D2R), anti-Glycogen synthase kinase 3 (p-GSK3-β) (Ser9), p-Akt (Ser473), anti-Glial fibrillary acidic protein beta (Anti-GFAP), anti-ionized calcium-binding adapter molecule 1 (anti-Iba-1), anti-phospho-c-Jun N-terminal kinase (p-JNK), anti-interleukin-1β (IL-1β), anti-caspase-3, anti-poly (ADP-ribose) polymerase-1 (Anti-PARP-1), anti-Bax, anti-Bcl-2 and anti-β-actin from Santa Cruz Biotechnology. Anti-beta actin was used as a loading control. The next day, the membranes were incubated with horseradish peroxidase-conjugated secondary antibodies diluted in 1×TBST for 1–2 h as appropriate; the immunoblots were developed using an ECL chemiluminescence system, according to the manufacturer’s instructions (Amersham Pharmacia Biotech, Uppsala, Sweden).

### 2.6. Brain Tissue Collection and Sample Preparation

For brain tissue collection, the mice were anesthetized and transcardially perfused with saline followed by (4%) paraformaldehyde and then fixed with (4%) paraformaldehyde for 48 h. Further, the brain tissues were immersed in a 20% sucrose solution for 48 h. Next, the Brain were fixed vertically in the OCT compound medium, Sakura Finetek USA, Inc., Torrance, CA, USA). For the brain cross-section (14 µm in size) using a vibratome (Leica, Nussloch, Germany) and stored at −80 °C.

### 2.7. Immunofluorescence Staining

The tissue slides were proceeded for immunofluorescence staining as described previously with minor modification [27,28]. Initially, the slides were dried at room temperature and washed twice with PBS 0.01 M solution for 8–10 min. The tissue slides were incubated in proteinase-K (5 min) and then washed twice for 5 min in PBS solution. Next, the protein was blocked for 1 h with 5% normal serum (goat/rabbit) D2R and 0.1% Triton X-100 in 0.01 M PBS solution. The tissue slides were then incubated with primary antibodies (1:100) ratio in 0.01 M PBS solution overnight at 4 °C. The following antibodies were used for the immunofluorescence detection; anti-p-GSK3-β (ser9), anti-p-Akta, anti-D2R, anti-IL1-β, anti-Caspase-3, anti-PSD-95, anti-SNAP-23, anti-ZO-1 anti-CD31. The tissue slides were then incubated for 2 h in the secondary antibody (1:100) fluorescein isothiocyanate (FITC), and tetramethylrhodamine isothiocyanates (TRITC) labeled secondary antibodies (anti-goat, anti-rabbit, and anti-mouse) from Santa Cruz Biotechnology. The 4′,6-diamidino-2-phenylindole (DAPI) was used for nucleus detection (8–10 min). The slides were covered with coverslips using with fluorescent mounting medium. Confocal laser scanning microscopy FluoViewer MPE-1000 (Olympus, Tokyo, Japan) was used to take the images and the maximum fluorescent intensity in the representative field was taken. The images were converted into Tiff format and the fluorescent intensity of the ipsilateral cortex and striatum region was measured and calculated via ImageJ win32 software (version 1.50, NIH, https://imagej.nih.gov/ij/, USA).

### 2.8. Assessment of Brain Lesion Volume

To measure lesion volume of the cortical area of TBI and TBI + quinpirole groups, the tissue slides were stained with cresyl violet and the images were taken with a simple light microscope and analyzed with ImageJ software. The injured areas of the TBI and TBI plus quinpirole groups were first outlined and then carefully calculated. The lesion volume was attained by multiplying the sum of the ipsilateral hemisphere area by the distance between the sections [24].

### 2.9. Nissl Staining

To analyze the neuronal cell death and lesion after brain injury, the Nissl staining was performed as described previously [24]. In brief, the slides were washed twice with 0.01 M PBS for 15 min followed by treatment with cresyl violet solution for another 1–15 min. The slides were washed with distilled water and dehydrated with ethanol (70%, 95%, and 100%). The tissue slides were cleared in xylene solution for 3 min and the mounting medium was added to the slides and coverslip was applied. A simple light microscope was used to examine the slides and taken images.

### 2.10. Cell Culture and Treatment

The Mouse hippocampal cell line HT22 and Microglial cell line BV2 were grown and maintained in Dulbecco’s modified Eagle medium (DMEM) medium (Thermo Fisher Scientific, Waltham, MA, USA) supplemented with 10% fetal bovine serum (Gibco, Grand Island, NY, USA). The final formulation comprises an additional 1% penicillin/streptomycin sulfate (Gibco, Grand Island, NY, USA). Cells were cultured in a humidified cell culture incubator equipped with a 5% carbon dioxide supply. Cell media was regularly replaced after every 2 days passaged. The cells were subjected to experimental procedures after confirmation of above 80% confluency.

Cell viability assay of mouse hippocampal neuronal HT22 cells was evaluated as described previously [29]. In brief, to know the effect of quinpirole the cells were cultured in 96 well plates (density of 1 × 10^4^ cells) containing Dulbecco’s modified Eagle’s medium (DMEM) 100 µL. After 24 h, the attached cells were subjected to microglial conditioned media (MCM). The cells were co-treated with three different concentrations of quinpirole (10 µM, 20 µM, and 40 µM) while the control cells were cultured only in DMEM (0.01%).

### 2.11. Microglial Conditioned Media

Mouse microglial cell line BV-2 was cultured to above 80% confluency were treated with Lipopolysaccharide (1 µg/mL) (Sigma-Aldrich, St. Louis, MO, USA) dissolved in Cell culturing media. After 24 h, media was aspirated and centrifuged to remove cells and debris. The clear supernatant was collected for further biochemical analysis.

### 2.12. Statistical Analysis

The western blot band’s results were scanned and analyzed by densitometry using sigma gel software (SPSS Inc., Chicago, IL, USA). ImageJ software (National Institutes of Health, Bethesda, MD, USA) was used for immunohistological analysis, and the obtained values were calculated as the mean ± S.E.M. The data analysis was performed by using one-way ANOVA followed by a post-hoc analysis of variance for control, TBI, and treated groups comparison. The data calculation and graphs were determined by using Prism 5 software (Graph Pad Software, Inc., San Diego, CA, USA). The statistical significance values were considered as *p* < 0.05. Note: * significantly different between control and brain injury, # significantly different between brain injury and quinpirole treated group.

## 3. Results

### 3.1. Quinpirole Regulated the D2R Expression Level in the Injured Brain and HT22 Cells

**Figure 1:**
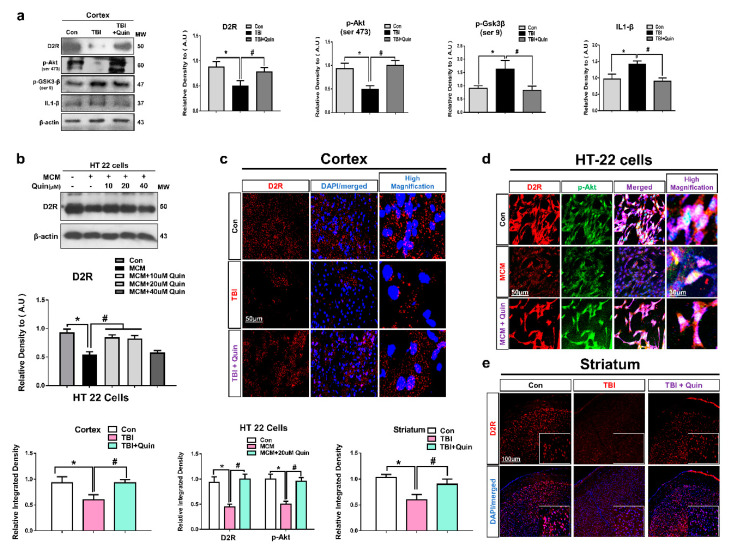
Quinpirole regulates the D2R/Akt/GSK3-β signaling pathway after brain injury. (**a**) Representative Western blot and histogram analysis of D2R, p-Akt, p-GSK3-β, and IL1-β in the ipsilateral cortex of an injured mouse brain. (**b**) Representing the Western blot analysis of D2R in HT22 cells. The β-actin was used as a loading control (*n* = 5). Western blot bands were quantified using the SigmaGel software. (**c**) Image showing results of immunofluorescence testing for D2R expression in the ipsilateral cortex of the control, brain injury, and quinpirole-treated mice groups. (**d**) D2R expression level and p-Akt co-localization in HT22 cells. (**e**) Image showing results of immunofluorescence testing for D2R expression in the ipsilateral straitum of the control, brain injury, and quinpirole-treated mice groups, with respective bar graphs (magnification ×10, *n* = 6). Data were obtained following three independent experiments. The ImageJ software was used for quantitative analysis of the confocal microscopy images and the maximum fluorescent intensity in the representative field was taken(green, FITC; red, TRITC; blue, DAPI). Values are represented as mean ± SEM. We performed the one-way ANOVA test followed by post-hoc analysis. A *p* value < 0.05 was considered statistically significant. * significantly different between control and brain injury groups, # significantly different between the brain injury and quinpirole-treated groups. ANOVA: analysis of variance, D2R: dopamine D2 receptors, GSK3-β: glycogen synthase kinase 3 beta, IL: interleukin, SEM: standard error of mean.

Many studies have reported that D2R agonist increases glial and neuronal cell D2R levels and suppresses the release of various inflammatory cytokines [30,31]. Studies have shown that quinpirole (a D2R agonist) activated D2R and suppressed neuroinflammation following brain injury in a mouse model of intracerebral hemorrhage (ICH) with Parkinson’s disease [19]. Based on this evidence, we performed Western blot and confocal microscopy analysis to investigate the effects of quinpirole on D2R expression levels especially in the ipsilateral cortex and striatum of brain-injured mice. Our results showed decreased D2R expression levels in the TBI experimental mice group. Notably, quinpirole treatment (1 mg/kg) significantly increased D2R expression in the quinpirole-treated group compared with the non-quinpirole-treated group of TBI mice (Figure 1a).

Deregulation of GSK3β is a critical step in the development and progression of neurodegenerative diseases via activation of neuroinflammatory processes [32]. Accumulating evidence suggests that the regulation of Akt and GSK3-β attenuates neurodegeneration and neuroinflamation [33]. Research has shown that D2R activation regulates the Akt and GSK3β protein levels [22]. Therefore, we performed Western blot analysis to determine the post-TBI expression levels of p-Akt and p-GSK3β and interleukin (IL)-1β. Our results showed increased expression levels of p-GSK3-β at (Ser 9) and IL-1β and decreased expression levels of p-Akt at (Ser 473) in the ipsilateral cortex of injured mouse brains. However, quinpirole treatment significantly regulated Akt/GSK3-β phosphorylation and reduced the IL-1β expression level in ipsilateral cortex of a damaged mouse brain (Figure 1a).

Furthermore, we investigated the protective role of quinpirole in vivo by in vitro studies. We subjected the HT22 cell line to Microglial conditioned media (MCM) treatment, and the cells were collected 24 h after MCM treatment. The HT22 cells were co-treated with three different concentrations of quinpirole (10 µM, 20 µM and 40 µM). Western blot analysis revealed that MCM-induced inflammatory mediators are associated with neuronal D2R deterioration. We observed that the administration of 10 µM or 20 µM reduced the toxic effect of MCM and significantly regulated D2R, which might be associated with the regulatory activity of p-Akt (Figure 1b).

**Figure 2:**
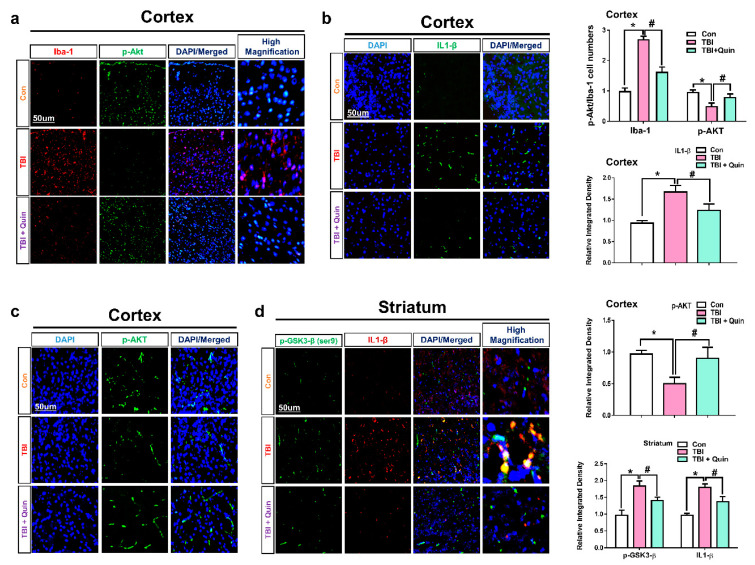
Quinpirole treatment reduces neuroinflammation via activation of the iba-1/p-Akt/p-GSK3-β and IL1-β signaling pathways after brain injury. (**a**) Double IF images of iba-1 and p-Akt in the ipsilateral cortex of brain-injured and quinpirole-treated mice. (**b**,**c**) Images showing results of immunofluorescence testing p-Akt (ser9) and IL1-β in the ipsilateral cortex after brain injury. (**d**) Images showing double immunofluorescence of p-GSK3-β (ser9) (FITC-label, green) and IL1-β (TRITC-label, red) (DAPI-label, blue) in the ipsilateral striatum with respective bar graphs, (magnification ×10, *n* = 6). Data were obtained after following three independent experiments. The ImageJ software was used for quantitative analysis of the confocal microscopy images and the maximum fluorescent intensity in the representative field was taken. Values are expressed as mean ± SEM. We performed the one-way ANOVA test followed by post-hoc analysis. A *p* value < 0.05 was considered statistically significant. * significantly different between control and brain injury groups, # significantly different between the brain injury and quinpirole-treated groups. ANOVA: analysis of variance, FITC: fluorescein isothiocyanate, GSK3-β: glycogen synthase kinase 3 beta, IL: interleukin, SEM: standard error of mean, TRITC: tetramethylrhodamine-isothiocyanate.

These results were validated by confocal microscopy. Our results also showed that the immunoreactivity of D2R was lower in the TBI group than in the control group. In contrast, quinpirole treatment improved D2R expression levels in the ipsilateral cortex and striatum after TBI (Figure 1c,e). Co-localization analysis of Akt and D2R revealed that their expression was significantly lesser in the MCM-treated HT22 cells. In contrast, quinpirole at a dose of 20 µM activated D2R and significantly increased Akt expression in HT22 cells (Figure 1d). Next, The p-Akt expression level was analyzed with iba-1, the double Immunofluorescence test result indicated the significantly reduced expression level of p-Akt and significantly increased expression of Iba-1 in the ipsilateral cortex of TBI mouse brain. However, post-TBI quinpirole treatment reversed this effect and significantly regulated the expression level of these markers in the ipsilateral cortex as compared to the TBI group of mice on day 7 (Figure 2a). We also evaluated Akt and IL1-β expression levels, particularly in the ipsilateral cortex, and confocal microscopy analysis revealed that post-TBI quinpirole treatment significantly regulated the expression level of these markers (Figure 2b,c). Moreover, co-localization analysis of p-GSK3β (Ser 9) and IL1-β showed increased expression levels in the ipsilateral striatum of TBI mice and also confirmed that post-TBI quinpirole treatment significantly reduced p-GSK3β and IL1-β expression levels (Figure 2d). Overall, these results confirm that post-TBI quinpirole administration may protects against neurodegenerative conditions via regulation of the D2R/Akt/GSK3β and IL-1β signaling pathways.

### 3.2. Quinpirole Reduced Gloisis and Atttenates D2R/Akt Level after Brain Injury

**Figure 3:**
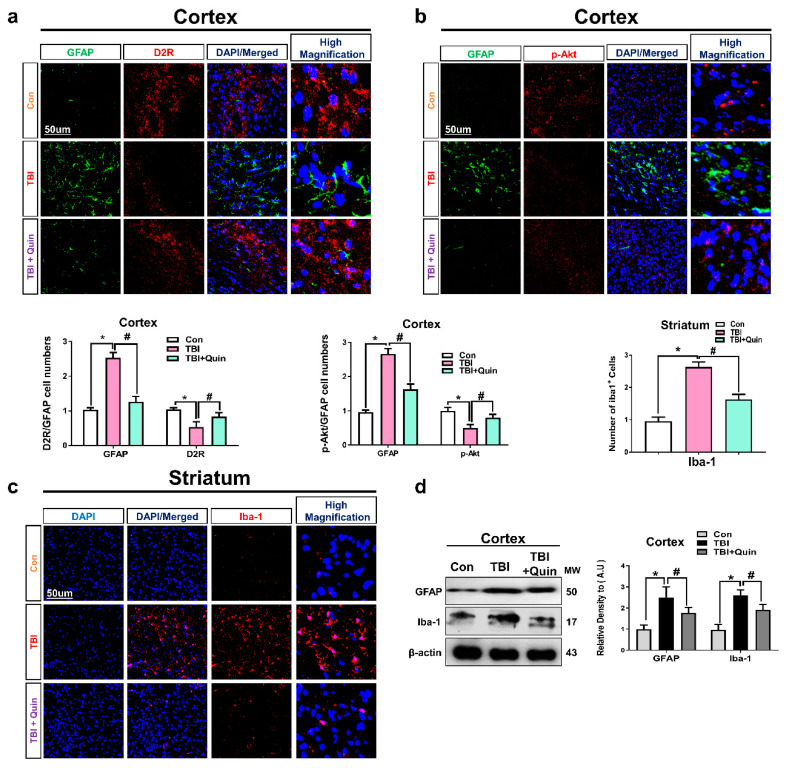
Quinpirole reduces astrocyte and microglia activation after brain injury. (**a**) Representative confocal microscopy images showing double immunoreactivity of GFAP and D2R expression level in ipsilateral cortex of TBI mouse model. (**b**) Images of double immunoreactivity of GFAP and p-Akt expression level in ipsilateral cortex of TBI mouse model (green, FITC; red, TRITC; blue, DAPI). (**c**) Confocal images of Iba-1 in ipsilateral striatum of brain-injured and quinpirole-treated mice, with respective bar graphs, (magnification ×10, *n* = 6). (**d**) Images of Western blot and histogram analysis showing GFAP and Iba-1 expression levels in ipsilateral cortex of brain-injured and quinpirole-treated mice. The β-actin was used as a loading control (*n* = 5). The ImageJ software was used for immunohistological analysis and the number of GFAP and iba-1 cells were quantified that containing D2R and p-Akt in the representative field. Data were obtained following three independent experiments. The ImageJ software was used for quantitative analysis of the confocal microscopy images. Values are expressed as mean ± SEM. We performed the one-way ANOVA test followed by post-hoc analysis. A *p* value < 0.05 was considered statistically significant. * significantly different between the control and brain injury groups, # significantly different between the brain injury and quinpirole-treated groups. ANOVA: analysis of variance, GFAP: glial fibrillary acidic protein, Iba-1: ionized calcium binding adaptor molecule 1, SEM: standard error of mean.

Gliosis plays an important role in the release of pro-inflammatory cytokines, such as IL-1β and tumor necrosis factor (TNF)-α and is a prominent feature of neurodegenerative conditions. Studies have reported that brain injury results in astrocyte and microglial activation, which precipitates further deleterious effects through the release of neuroinflammatory mediators [34,35,36]. Reportedly, D2R agonists are shown to significantly reduce the activation of astrocytes and the release of TNF-α in the spinal cord of a mouse model of amyotrophic lateral sclerosis and also prevent motor neuron loss (or death). Previously, studies reported the suppression of microglia following D2R activation [37]. While another study was also well suggested that Akt and GSK3β plays an essential role in glial response [38]. Based on these reports, we investigated whether quinpirole treatment could inhibit neuroinflammatory responses in our mouse model of TBI. Therefore, we evaluate glial fibrillary acidic protein (GFAP); a marker of active astrocytes, ionized calcium-binding adaptor molecule 1 (Iba-1); a marker of active microglia together with D2R and p-AKT expression level in ipsilateral or striatum after brain injury. Our double Immunofluorescence test results showed significantly increased immunoreactivity of GFAP and the expression level of D2R and p-AKT was significantly decreased in the ipsilateral cortex of TBI group as compared to saline treated group of mice. However, post-TBI quinpirole treatment reversed this effect, and significantly regulated the expression level of these markers on day 7 (Figure 3a,b). Moreover, we also checked the expression level of iba-1 in the ipsilateral striatum of TBI group of mice. Confocal microscopy result for iba-1 showed the significantly increased expression level of iba-1 in the TBI group of mice. However, the expression level of iba-1 was significantly decreased in quinpirole-treated TBI mice on day 7 (Figure 3c). Interestingly, the results of the Western blot analysis also showed increased Iba-1 and GFAP expression levels, indicating that the number of activated microglia and astrocytes was higher in TBI mice than in mice treated with saline (Figure 3d). Moreover, glial cell activation was significantly lower in the quinpirole-treated group than in the TBI group. Our results show that quinpirole treatment potentially ameliorates TBI-induced glial cell activation on day 7. This condensation of glial cells may be associated with astrocyte and microglial D2R and p-Akt modulation following quinpirole treatment.

### 3.3. Quinpirole Reduced Neuronal Apoptosis after Brain Injury

**Figure 4:**
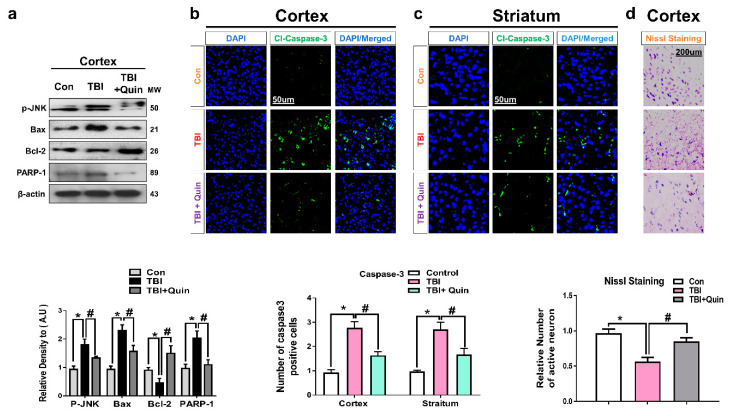
Quinpirole inhibits brain injury-induced neuronal apoptosis in mice brain. (**a**) Representative images showing results of immunoblot and histogram analysis of p-JNK, Bax, Bcl-2, and PARP-1 proteins in the ipsilateral cortex of injured mouse brain. The β-actin was used as a loading control (*n* = 5). (**b**,**c**) Immunofluorescence test images showing cl-caspase-3 immunoreactivity in the ipsilateral cortex and striatum of injured mouse brain, (green, FITC; blue, DAPI) with respective bar graphs, (magnification ×10, *n* = 6). (**d**) Nissl stain images of the ipsilateral cortex. ImageJ software was used for immunohistological analysis. Data were obtained following three independent experiments. The ImageJ software was used for quantitative analysis of the nissl images and confocal microscopy images. The integrative density of the number of caspase3 positive cells were quantified in the representative field. Values are expressed as mean ± SEM. We performed the one-way ANOVA test followed by post-hoc analysis. A *p* value < 0.05 was considered statistically significant. * significantly different between the control and brain injury groups, # significantly different between the brain injury and quinpirole-treated groups. ANOVA: analysis of variance, SEM: standard error of mean.

Many studies performed in a TBI mouse model have reported neuronal apoptosis after brain injury, particularly in the perilesional areas and striatum [39,40]. Using Western blot analysis, we investigated apoptotic markers, including Bax, Bcl-2, and PARP1 in the ipsilateral cortex (Figure 4a). We observed that compared with saline-treated mice, brain-injured mice showed a marked increase in neuronal apoptosis. Interestingly, we found significantly lower levels of p-JNK and apoptotic markers in the ipsilateral cortex in the quinpirole-treated group than in the non-quinpirole-treated group. These results were further validated by confocal microscopy. Immunofluorescence test results revealed increased expression of caspase-3 ipsilateral cortex and striatum in brain-injured mice.

Additionally, compared with the TBI group, the quinpirole-treated group showed a significant reduction in the high expression of caspase-3 in the ipsilateral cortex and striatum (Figure 4b,c). We performed Nissl staining to further assess neuronal cell death; compared with the control group, the brain-injured mice group showed a reduced number of surviving neurons in the ipsilateral cortex. Notably, quinpirole treatment reversed this effect and significantly increased the number of surviving neurons in the ipsilateral cortex of quinpirole-treated TBI mice (Figure 4d). These results suggest that the impact of brain injury extend to the ipsilateral cortex and striatum, and quinpirole treatment is known to inhibit neuronal apoptosis possibly via D2R activation in ipsilateral side of injured mouse brain.

### 3.4. Quinpirole-Induced Restoration of Blood–Brain Barrier Disruption and Lesion Volume after Brain Injury

**Figure 5:**
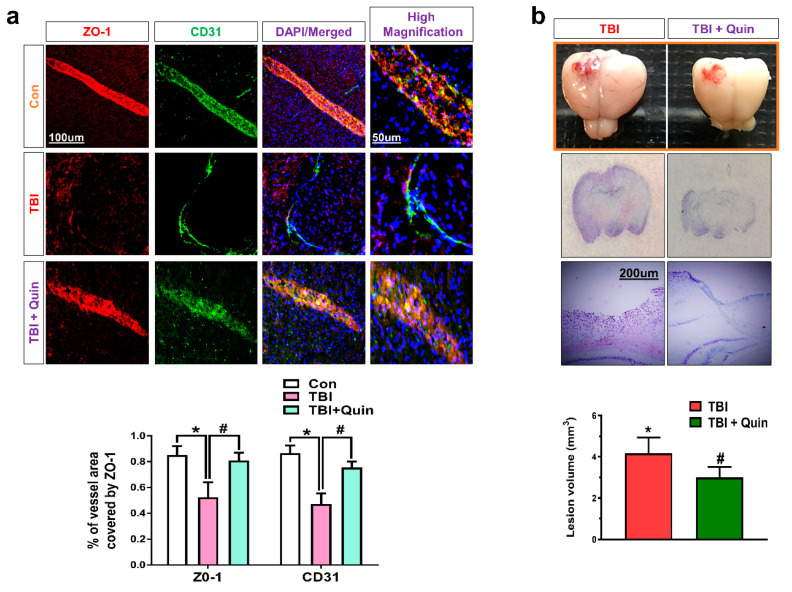
Quinpirole regulates the BBB-associated ZO-1 and CD31 expression levels and lesion volume after brain injury. (**a**) Representative confocal microscopy images for ZO-1 (TRITC-label, red) and CD31 (FITC-label, green) immunofluorescence reactivity in the ipsilateral cortex in injured mouse brain (green, FITC; red, TRITC; blue, DAPI). (**b**) Representative images showing TBI mouse brain after surgery, and Nissl-stained images showing the lesion volume in the brain injury and quinpirole-treated groups, with respective bar graphs, (magnification ×10, *n* = 6). Data were obtained following three independent experiments. The ImageJ software was used for quantitative analysis of the confocal microscopy images and the percentage of vessels were quantified that containing ZO-1 in the representative field. Values are expressed as mean ± SEM. We performed the one-way ANOVA test followed by post-hoc analysis. A *p* value < 0.05 was considered statistically significant. * significantly different between the control and brain injury groups, # significantly different between the brain injury and quinpirole-treated groups. ANOVA: analysis of variance, BBB: blood–brain barrier, CD31: cluster of differentiation 31, FITC: fluorescein isothiocyanate, SEM: standard error of mean, TRITC: tetramethylrhodamine-isothiocyanate, ZO-1: zonula occludens-1.

Previous research has shown that TBI results in severe BBB disruption, which invariably leads to severe complications in the affected areas [41]. Activation of astrocytic signaling causes BBB injury through the release of cytokines or chemokines and immune cell recruitment. Therefore, we evaluated the BBB breakdown and the possible role of quinpirole in the restoration of the disrupted BBB in our TBI mouse model. On confocal microscopy, co-localization of zonula occludens-1 (ZO-1) and a cluster of differentiation 31 (CD31) proteins showed that compared with the saline-treated control mice, the brain-injured mice showed significantly decreased ZO-1 expression levels in endothelial cells, and compared with brain-injured mice, the quinpirole-treated mice showed elevation of the reduced ZO-1 protein levels and a significant increase in its expression on post-TBI day 7 (Figure 5a). It is well known that brain injury immediately causes gross tissue disruption at the site of injury. Therefore, we also assessed the lesion volume on post-TBI day 7. Histopathological examination of specimens obtained from brain-injured mice showed a marked increase in the contusion and lesion volume in this group, which was significantly reduced following quinpirole treatment (Figure 5b).

### 3.5. Quinpirole Attenuated Synaptic Dysfunction after Brain Injury

**Figure 6:**
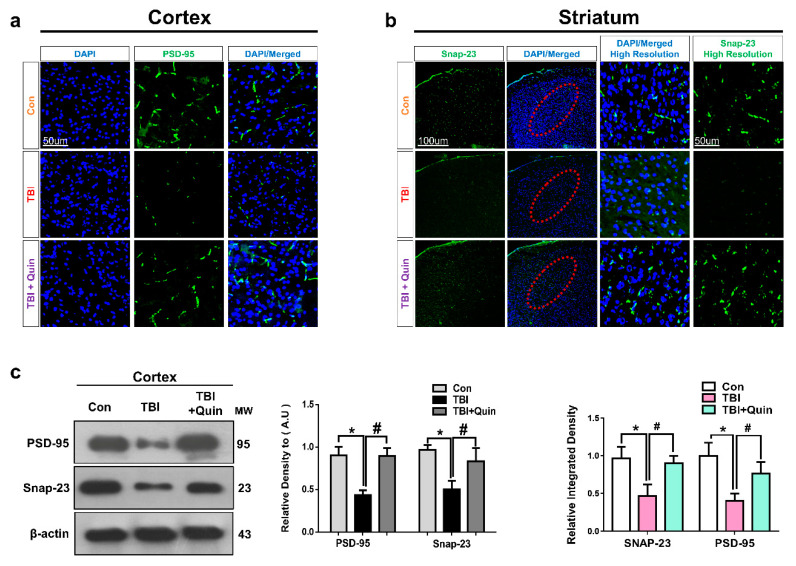
Quinpirole regulates synaptic protein loss after brain injury. (**a**,**b**) Confocal microscopy images for PSD-95 and SNAP-23 expression in the ipsilateral cortex and striatum of an injured mouse brain (green, FITC; blue, DAPI), with respective bar graphs, the red dotted lines showing the striatum region, (magnification ×10, *n* = 6). The protein band levels were quantified using the SigmaGel software. (**c**) Images showing results of Western blot and histogram analysis for PSD-95 and sanp-23 in ipsilateral cortex of injured mouse brain. The β-actin was used as a loading control (*n* = 5). Data were obtained following three independent experiments. The ImageJ software was used for quantitative analysis of the confocal microscopy images. Values are expressed as mean ± SEM. We performed the one-way ANOVA test followed by post-hoc analysis. A *p* value < 0.05 was considered statistically significant. * significantly different between the control and brain injury groups, # significantly different between the brain injury and quinpirole-treated groups. ANOVA: analysis of variance, PSD-95: post-synaptic density protein 95, SEM: standard error of mean, SNAP-23: synaptosomal-associated protein 23, TBI: traumatic brain injury.

Previous studies have reported that brain injury causes synaptic protein loss, which leads to memory impairment [35,42]. Therefore, we evaluated the expression levels of synaptic proteins, including synaptosomal-associated protein 23 (SNAP-23) and post-synaptic density protein 95 (PSD-95) in our TBI mouse model. We performed confocal microscopy for PSD-95 and SNAP-23 in ipsilateral cortex and striatum respectively. We observed that brain injury significantly decreases synaptic protein expression, whereas quinpirole treatment significantly increased synaptic protein loss in an injured mouse brain (Figure 6a,b). Western blot analysis revealed that compared with saline-treated mice, brain-injured mice showed reduced PSD-95 and SNAP-23 expression in the ipsilateral cortex (Figure 6c). However, quinpirole treatment significantly restored synaptic proteins following brain injury.

## 4. Discussion

An optimal therapeutic approach to brain injuries is unavailable owing to the multifactorial pathogenesis of brain trauma. Brain injuries lead to cognitive dysfunction that can be prevented by DA therapies targeted at the restoration of cognitive impairment [43,44]. The most important neurotransmitters in the central nervous system: glutamate is released from multiple stores after a TBI and the activation of D2Rs could contribute in the modulation of the glutamate release both from neurons than from astrocytes. Hence, activation of D2Rs may play essential role after TBI-induce disturbance in neurotransmitters. In this study, we observed that quinpirole (a D2R agonist) plays a significant role in brain injury-induced neuroinflammation, neurodegeneration, and synaptic dysfunction. We focused on the neuroprotective effect of quinpirole following brain injury in mice. This report shows that post-TBI quinpirole administration attenuates several neuropathological events, such as glial cell activation, neuroinflammation, neuronal apoptosis, and synaptic dysfunction via the D2R/Akt GSK3β/IL-1β signaling pathways. Since the TBI-induced striatal glial activation and expression of pro-inflammatory cytokines and therapeutic potential of D2R activation in the ipsilateral striatum is mostly known previously as compared to the ipsilateral cortex; thus, still it is essential to investigate the therapeutic potential of D2R in the cortex of TBI mouse brain [45]. Therefore, we investigated the neuroprotective effect of D2R activation mainly in the ipsilateral cortex, while we checked slightly the ipsilateral striatum of the injured mouse brain. Our results suggest that quinpirole activates D2R, which plays a crucial role in several neuropathological events particularly in ipsilateral cortex after brain injury.

Brain injury leads to neuroinflammation, which contributes to severe neurodegeneration. Studies have reported chronic neuroinflammatory responses in the cortex and hippocampus of an injured mouse brain [46,47]. In our TBI mouse model, we observed increased neuroinflammation indicated by microglial and astrocyte activation in the ipsilateral cortex and striatum. Interestingly, post-TBI quinpirole treatment significantly reduced the increased gliosis and release of pro-inflammatory markers. Our results are consistent with those reported by previous studies [12]. It is known that the regulation of p-GSK3-β via p-Akt is involved in the cell survival pathway [48]. A previous study showed that injury-induced disruption of Akt and GSK3β expression in glial cells is a major contributor to the mechanistic of glial cell adaptation as well as protection in response to cell damage. Thus Akt and GSK3β play an essential role in glial response and excitotoxic lesion outcome of injury [38]. Another study investigated the role of Akt/GSK3β pathway in acute brain injury after subarachnoid hemorrhage [49,50]. The regulation of GSK3-β and Akt via D2R could be a novel therapeutic approach following brain injury. In the present study, we investigated the protective effect of quinpirole mediated via the D2R/GSK3β/Akt signaling pathway. We found decreased expression of D2R/Akt and increased expression of p-GSK3-β and IL-1β after TBI, based on Western blot and immunofluorescence analysis. However, these levels normalized to the baseline levels in quinpirole-treated mice. A previous study also reported the anti-neuroinflammatory effect of quinpirole via D2R activation in an ICH injury model [19]. The BBB plays a central role in brain homeostasis. However, BBB disruption leads to enhanced cytokine infiltration and neuronal susceptibility.

Several tight junction proteins, including claudin, occludin, and ZO-1, are essential for the maintenance of BBB integrity [51]. BBB breakdown following brain injury is attributable to significant histopathological alterations and tissue loss in the affected areas [52]. Double immunofluorescence staining performed for ZO-1 and CD31 showed significantly low levels of these proteins in the ipsilateral cortex of an injured mouse brain. Notably, quinpirole treatment restored ZO-1 and CD31 levels in the ipsilateral cortex of an injured mouse brain. Quinpirole-regulated restoration of the disrupted BBB is attributable to reduced neuroinflammation and active gliosis. A previous study has reported that brain injury is strongly associated with deregulated tight junction proteins [53]. Brain injury is known to cause marked tissue disruption [34]. We observed increased lesion volume in an injured mouse brain, and that quinpirole treatment significantly reduced the lesion volume, which suggests that quinpirole aids in the repair of the brain after injury and restores tight junction proteins to inhibit infiltration of cytokines and other blood-borne biochemical agents. Moreover, we observed a significant increase in the contusion volume after brain injury, indicating that severe damage is associated with tissue disruption in the ipsilateral cortex of injured mouse brain. Notably, all these effects were ameliorated in brain-injured mice that received quinpirole treatment.

Increasing evidence has shown neuronal apoptosis after brain injury [54]. Our results showed increased expression of neuronal apoptotic markers, including Bax and PARP1, and decreased expression of Bcl-2, an anti-apoptotic protein. Quinpirole treatment significantly reduced the increased levels of pro-apoptotic and increase the reduced level of anti-apoptotic markers in the ipsilateral cortex of brain-injured mice. A previous study supports the protective role of D2/D3 receptor agonist ropinirole protects against apoptosis-induced neurodegeneration via a JNK-dependent pathway [11]. Our results are consistent with those of a previous study in which D2R agonists were shown to reduce neuronal apoptosis [55]. In accordance with the caspase3 result, the nissl staining results also showed the significantly increase number of apoptotic and degenerated neurons in the ipsilateral side of TBI mouse brain as compared to saline- treated control group of mice. However, the number of damage and degenerated neuronal cells were significantly reduced in quinpirole-treated group of mice after TBI on day 7.

Synaptic protein loss is associated with brain injury and leads to cognitive deficits and impaired neurotransmission. A study has reported that DR activation protects against amyloid-β oligomer-mediated synaptic dysfunction. Therefore, we evaluated synaptic protein markers after brain injury. The results of Western blot and immunofluorescence testing showed that quinpirole treatment reversed the deregulated levels of synaptic protein markers, including PSD-95 and SNAP-23 in TBI mice. Moreover, other studies have also reported that D2R is vital for several brain functions, including learning and working memory. We concluded that quinpirole could be a potentially useful therapeutic agent to restore synaptic function after brain injury and to improve the cognitive performance of brain-injured mice.

These results suggest that brain injury may cause D2R suppression, which consequently activates deleterious signaling pathways at a later stage after brain injury, and that quinpirole-mediated D2R activation produces a neuroprotective effect in the ipsilateral cortex and striatum of injured brains.

## 5. Conclusions

**Figure 7:**
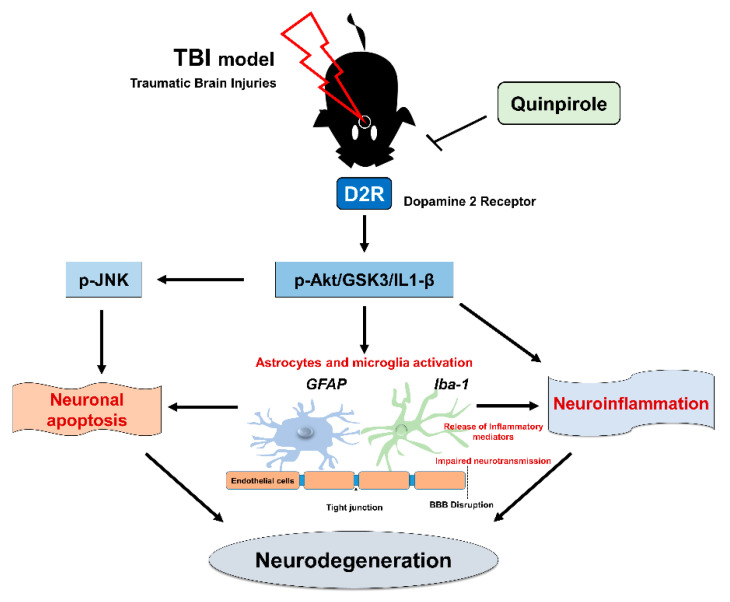
Schematic representation of the proposed mechanism of neuroprotection of quinpirole against brain injury-induced neuroinflammation, BBB disruption and neurodegeneration via D2R and Akt/GSK3-β/IL-1β signaling in the injured mouse brains.

This study highlights the significant role of D2R in neurodegenerative conditions affecting the ipsilateral cortex after brain injury and that D2R regulation might be an effective therapeutic strategy to inhibit glial cell-induced neuroinflammation in a mouse model of brain injury (Figure 7). In this study, we discuss the role of quinpirole (a D2R agonist), that can potentially attenuate several neuropathological processes via D2R/Akt/GSK3-β/IL-1β signaling in the ipsilateral cortex and striatum of an injured mouse brain. Further studies are warranted to gain a deeper understanding of the molecular mechanisms contributing to the neuroprotective effects of quinpirole via D2R activation in neuropathological events associated with brain injury.

## Data Availability

The authors hereby declares that the data presented in this study will be presented upon request from the corresponding author.
